# Anticoagulant Activity of Heparins from Different Animal Sources are Driven by a Synergistic Combination of Physical-chemical Factors

**DOI:** 10.1055/a-1946-0325

**Published:** 2022-10-11

**Authors:** Stephan N.M.C.G. Oliveira, Ana M.F. Tovar, Francisco F. Bezerra, Adriana A. Piquet, Nina V. Capillé, Paloma S. Santos, Eduardo Vilanova, Paulo A.S. Mourão

**Affiliations:** 1 Laboratório de Tecido Conjuntivo, Hospital Universitário Clementino Fraga Filho and Instituto de Bioquímica Médica Leopoldo de Meis, Universidade Federal do Rio de Janeiro, Rio de Janeiro, Brazil

**Keywords:** anticoagulant, heparin, low-molecular-weight heparin, physical-chemical concepts, thromboembolism

## Abstract

Heparin has already been found in a variety of animal tissues but only few of them became effective sources for production of pharmaceutical preparations. Here, we correlate physical-chemical features and anticoagulant activities of structurally similar heparins employed in the past (from bovine lung, HBL), in the present (from porcine intestine, HPI) and in development for future use (from ovine intestine, HOI). Although they indeed have similar composition, our physical-chemical analyses with different chromatography and spectrometric techniques show that both HOI and HBL have molecular size notably lower than HPI and that the proportions of some of their minor saccharide components can vary substantially. Measurements of anticoagulant activities with anti-FIIa and anti-FXa assays confirmed that HPI and HOI have potency similar each other but significantly higher than HBL. Such a lower activity of HBL has been attributed to its reduced molecular size. Considering that HOI also has reduced molecular size, we find that its increased anticoagulant potency might result from an improved affinity to antithrombin (three times higher than HBL) promoted by the high content of *N* ,3,6-trisulfated glucosamine units, which in turn are directly involved in the heparin-antithrombin binding. Therefore, the anticoagulant activity of different heparins is driven by a balance between different physical-chemical components, especially molecular size and fine-tuning composition. Although such minor but relevant chemical differences reinforce the concept that heparins from different animal sources should indeed be considered as distinct drugs, HOI could be approved for interchangeable use with the gold standard HPI and as a suitable start material for producing new LMWHs.

## Introduction

Heparin purified from mammalian tissues has been employed as prevalent anticoagulant drug for nearly 100 years, which makes it one of the oldest biologic medicines still available for clinical use today. 1 Although discovered in dog's liver in the early 20 ^th^ century, large-scale manufacturing of heparin was initially based on bovine lung (HBL); however, in the 1960's decade, HBL started to be gradually replaced by heparin preparations with improved activity and production yield sourced from porcine intestine mucosa (HPI), up to its definitive discontinuation in the 1990's. 2

Considering that manufacturing synthetic heparin through chemical or chemoenzymatic processes or metabolic engineering is still far from being a reality, its production will continue to be dependent on a vast supply of raw animal material for some time to come. 3 Currently, 99% of the unfractionated heparin (UFH) and all the low-molecular-weight heparin (LMWH) consumed worldwide are sourced from porcine intestine mucosa, with ∼80% of the production concentrated in China. 4 Such a supply chain based on a single animal source from a single country has shown to be insufficient to meet the growing worldwide demand and is susceptible to sudden shortages caused by porcine epidemics. 5 Different stakeholders have already pointed out that the only feasible way to reinforce the global supply of heparins is to disseminate the use of products from alternative animal sources, such as bovine intestine mucosa (HBI), which currently accounts for only 1% of the UFH consumed worldwide, and ovine intestine mucosa (HOI) in development as new UFH and LMWH products. 6

**Fig. 1:**
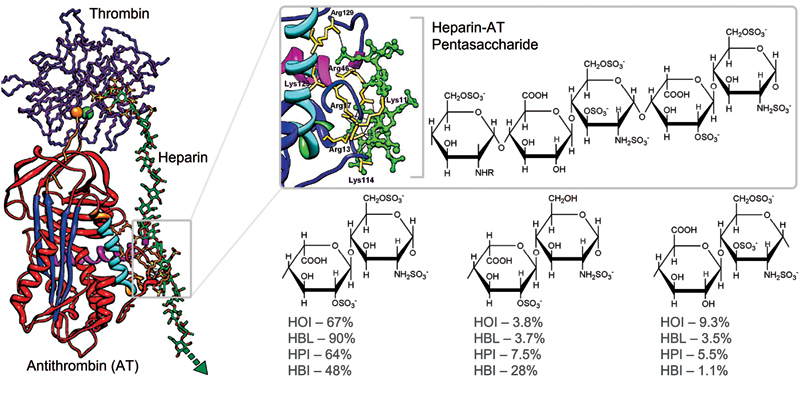
Heparin-antithrombin interaction and compositional differences among heparins produced from distinct animal sources. Interaction of heparin with antithrombin is mediated by a specific pentasaccharide sequence. Heparins from distinct animal sources, such as porcine (HPI), bovine (HBI) and ovine (HOI) mucosa and bovine lung (HBL), can present notable differences in the proportion of some of their disaccharide constituent units; for example: 2-sulfated α-L-iduronic acid 1→4 *N* ,6-disulfated α-D-glucosamine; 2-sulfated α-L-iduronic acid 1→4 *N* -sulfated α-D-glucosamine and β-D-glucuronic acid 1→4 *N* ,3,6-trisulfated α-D-glucosamine.

Heparin is a sulfated polysaccharide mostly composed of alternating units of 2- sulfated α-L-iduronic acid and *N* ,6-disulfated α-D-glucosamine but bearing several minor modifications in both *N-* / *O* -sulfation and *N* -acetylation sites, as well as substitutions by β-D-glucuronic acid units. 7 Such minor structural variations in combination with physical-chemical factors like molecular mass and anionic strength entail great impact in the biologic properties of heparin. 8 For example, the remarkable anticoagulant activity of heparin mostly relies on the inactivation of activated thrombin (FIIa) and activated factor X (FXa) by potentiating antithrombin (AT) through its binding with a specific pentasaccharide sequence containing several modifications ( Fig. 1 ). 9

Although heparins obtained from different animal sources present as major component the same trisulfated disaccharide unit, their minor components may vary considerably, as broadly reported in the literature ( Fig. 1 ). 10 11 12 Among these differences, we highlight the increased proportion of 6-desulfated α-D-glucosamine units in HBI in comparison with other heparins preponderantly composed of disaccharides containing *N* ,6-disulfated α-D-glucosamine. 13 Such a high 6-desulfation along with the small quantity of *N* ,3,6-trisulfated α-D-glucosamine units, which is an essential component of the AT-binding pentasaccharide sequence, are attributed to the reduced anticoagulant potency of HBI in comparison to HPI (120 and 180 International Units [IU]/mg). 14

Although the reduced potency of HBI can be clearly correlated to their extensive compositional differences, the anticoagulant activities of distinct heparins bearing similar disaccharide composition may also present considerable variations, which in turn must also be driven by other physical-chemical factors, such as, for example, the reduced potency of HBL attributed to its diminished molecular size. 15 In the present work, we evaluated the anticoagulant properties, structure and physical-chemical characteristics of distinct pharmaceutical heparins sharing similar disaccharide composition employed in the past (HBL), in the present (HPI) and in development for future use (HOI). To detect minor compositional differences and determine the physical-chemical features of these heparins in further detail, we employed a variety of state-of-the-art chromatography and spectrometric techniques, which were then correlated to their anticoagulant potencies measured with in vitro assays and antithrombotic activities determined by an in vivo model of venous thrombosis in mice. We find that differences in the molecular size in combination with discrete variations in the quantity and distribution of minor components have a direct impact in the ability of these heparins to bind with AT and, consequently, in their different anticoagulant potencies. Besides demonstrating that the anticoagulant activity of heparins is modulated by a fine-tuning combination of physical-chemical components, we deepen the understanding that heparins from different animal sources, even those sharing similar compositions and anticoagulant potencies, could present minor but relevant chemical and/or pharmacological differences, and thus should be considered as distinct drugs, even when approved for interchangeable use or as start material for producing new LMWHs.

## Materials and Methods

### Heparins and Standards

Batches of pharmaceutical grade HOI (3) and HBL (1) were kindly given by Dr. Walter Jeske and Dr. Barbara Mulloy, respectively, and commercial HPI formulations (Hemofol™ and Parinex ^TM^ , four batches of each) were obtained from Cristália (Itapira, SP, Brazil) and Hipolabor (Sabará, MG, Brazil), respectively. The 6 ^th^ International Heparin Standard (2,145 units per vial, Lot No. 07/328) and the 3 ^th^ International Standard for LMWH (Lot 11/176) were obtained from the National Institute for Biological Standards and Control (NIBSC; Potters Bar, United Kingdom) and the heparin sodium molecular-weight calibrant (Lot No. FOL4830) from USP (Rockville, U.S). Dermatan sulfate and oversulfated chondroitin sulfate (Lot No. HOM191) standards were purchased from Sigma-Aldrich (St. Louis, U.S.) and USP, respectively.

### Analytical Anion-exchange Chromatography

Anionic-strength of the heparins were assessed via analytical anion-exchange chromatography using an IonPac AS11-HC column (Dionex; Sunnyvale, CA, U.S.) equilibrated with 10 mM Tris-HCl, 0.4 M NaCl (pH 7.4), linked to a HPLC system (Shimadzu; Tokyo, Japan), as described elsewhere. 13 HOI, HBL and HPI (200 μg of each), dermatan sulfate (30 μg) and oversulfated chondroitin sulfate (50 μg) were applied into the column, washed with equilibration buffer (10 mL) and then eluted with a flow rate of 0.5 mL min ^−1^ through a linear gradient of 0.4→2.5 M NaCl (40 mL). Chromatograms were acquired by monitoring the U.V. absorbance (215 nM) subtracted from their respective backgrounds. To check the homogeneity of the heparins, 5 mg of each HOI, HBL, HPI and HBI were applied into a Fractogel EMD TMAE Hicap (Sigma-Aldrich), linked to a HPLC system (Shimadzu), equilibrated with 20 mM Tris-HCl, 1 mM EDTA (pH 7.4). Heparins were eluted with a flow rate of 1.0 mL min ^−1^ through a step-wise gradient of equilibration buffer supplemented with 16.7% (5 minute) →60% (20 minute) →100% (10 minute) 2 M NaCl continuously monitored by U.V. absorbance (215 nM).

### Analytical Size-exclusion Chromatography

Molecular-weight distribution of the heparins were determined through analytical gel-permeation chromatography using a set of TSK gel G4000 SW + G3000 SW columns (Tosoh; Tokyo, Japan), linked to a HPLC system (Shimadzu), equilibrated with 0.1 M ammonium acetate as previously described. 16 HOI, HBL and HPI (200 μg of each) were eluted at 0.3 mL min ^−1^ flow rate at 40°C and monitored by differential refractive index. The columns were calibrated using heparin sodium molecular-weight calibrant from USP. Weight average molecular weight ( *Mw* ), number average molecular weight ( *Mn* ) and polydispersity degree (PD) were calculated as described elsewhere. 13

### Solution NMR Spectrometry Analyses

NMR spectra of the heparins were recorded using a DRX 800 MHz Spectrometer (Bruker; Billerica, U.S.) with triple-resonance probe as previously described. 13 About 20 mg of each HOI, HBL and HPI were dissolved in 0.5 mL 99.9% deuterium oxide (Cambridge Isotope Laboratory; Cambridge, U.S) and then the spectra were recorded at 35°C with HOD (deuterium oxide) suppression by pre-saturation. 1D ^1^ H NMR spectra were recorded with 32 scans. Phase-sensitive ^1^ H- ^1^ H MLEV17 TOCSY spectra (4046 × 400 points) were acquired with spin-lock field of 10 kHz and mix time of 80 milliseconds. ^13^ C/ ^1^ H multiplicity-edited HSQC spectra (1024 × 256 points) were acquired with globally optimized alternating phase rectangular pulses for decoupling (GARP). ^1^ H and ^13^ C chemical shifts were calibrated (0 ppm) with basis on signals from external standards trimethylsilyl propionic acid and methanol, (both from Sigma-Aldrich), respectively. Spectra were processed using the software Top-Spin 4.0 (Bruker).

### Disaccharide Analyses with SAX-HPLC Chromatography

Heparin disaccharides were produced by incubating samples (200 µL) containing 5 mg of each HOI, HBL and HPI with a mixture containing 20 U mL ^-1^ of heparinases I, II and III (all from *Flavobacterium heparinum* ; Sigma-Aldrich) in 20 mM Tris HCl for 24 hours at 37°C. The products of these incubations (20 µL of each) and a mixture of heparin disaccharide standards were applied into a SUPELCOSIL SAX1 column (Sigma-Aldrich), coupled to an HPLC system (Shimadzu), and then eluted at 0.2 mL min ^-1^ through a gradient of 0→1.0 M NaCl in deionized water containing 0.1 M HCl continuously monitored by U.V. absorbance (232 nM). The retention times and peak integrals of the disaccharides were calculated using the HPLC software (Shimadzu).

### Oligosaccharides Analyses with Size-exclusion Chromatography

Oligosaccharides were produced by incubating samples (200 µL) containing 5 mg of each HOI, HBL and HPI with 10 µL of heparinase I (250 U mL ^-1^ ; Sigma-Aldrich) in 20 mM Tris HCl for 24 hours at 37°C. The incubations' products and the 3 ^th^ International Standard for LMWH (20 µL of each) were applied into TSK G-3000 + TSK G-2000 (Tosoh) columns coupled in series to an HPLC system (Shimatzu), and then eluted at 0.3 mL min ^-1^ with 0.1 M ammonium acetate (pH 6.0) continuously monitored by refractive index. Retention times and peak integrals of the oligosaccharides were determined using the HPLC software (Shimadzu).

### Fluorimetric Analyses of Heparin-antithrombin Interactions

Affinities of the heparins with antithrombin (AT) were evaluated with basis on dissociation constants ( *K _d_* ) calculated through the fluorescence gain promoted by their binding using a Varian Cary Eclipse spectrofluorometer (Varian, Palo Alto, U.S.), as previously described. 13 Briefly, solutions of AT (0.5 μM) in 600 μL phosphate buffer (20 mM sodium phosphate, 0.1 M NaCl, 0.1 mM EDTA and 0.1% polyethylene glycol 8,000, pH 7.4) were titrated with aliquots (0.2 μL) from each HOI, HBL and HPI (2.5 mg mL ^-1^ stock solution) and then incubated (1 minute) in quartz cuvettes. Changes in the intrinsic fluorescence spectrum of AT were monitored from 300 to 450 nm, with excitation wavelength set to 280 nm. All the spectra were acquired at 37°C, continuous stirring and bandwidths set to 5 nm for excitation and 10 nm for emission. Dissociation constants ( *K _d_* ) for the heparin-AT bindings were calculated with basis on the enhancements of tryptophan fluorescence emissions by nonlinear regression, as described elsewhere. 13 Spectral areas were calculated using Cary Eclipse Software Patch (Varian).

### Activated Partial Thromboplastin Time Assay (APTT)

Human plasma (100 μL) and different concentrations of HOI, HBL and HPI were incubated for 2 minute at 37°C with 100 μL APTT reagent (kaolin bovine phospholipid from Biolab-Merieux AS; Rio de Janeiro, Brazil). After incubation, 100 μL CaCl _2_ (25 mM) was added to the mixture and then the clotting time was recorded in an Amelung KC4A coagulometer (Heinrich Amelung GmbH; Lemgo, Germany). The results were expressed as the ratio of clotting time in the presence (T) and absence (T _0_ ) of different volumes (2→10 μL) of the heparins samples (50 μg mL ^−1^ ) and then fitted as second order polynomial curves. Anticoagulant potencies as IU mg ^−1^ (dry weight) were calculated with basis on values obtained fitting the 6 ^th^ International Heparin Standard curve (10 IU mL ^−1^ ). 16 Anticoagulant potencies of the heparins were compared by ANOVA with Bonferroni post-hoc test using Origin 8.0 software (OriginLab; Northampton, U.S.).

### In vitro anti-FXa and Anti-FIIa Activities

The heparins were subjected to FIIa or FXa amidolytic activity assessments by measuring the hydrolysis of chromogenic substrates as previously described. 13 AT (10 nM) from Hematologic Technologies (Essex Junction, U.S.) and 0→0.4 μg mL ^−1^ of the heparins (dry weight) were incubated in TS/PEG buffer (0.02 M Tris/HCl, 0.15 M NaCl and 1.0 mg mL ^−1^ polyethylene glycol 8,000, pH 7.4) and then 2 nM FXa or FIIa (Hematologic Technologies) was added to trigger the reaction. After incubation for 60 second at 37°C, residual FXa or FIIa activities were determined by adding 100 μM of chromogenic substrates S-2765 or S-2238, respectively (Chromogenix; Molndal, Sweden) followed by recording absorbance (405 nm) during 300 second in a Thermomax Microplate Reader (American Devices; Sunnyvale, U.S.). Anti-FXa and anti-FIIa activities were calculated with basis on parallel line assays performed with the 6 ^th^ International Heparin Standard using SoftMax Pro 5.4.1 software (American Devices). The anti-FXa and -FIIa activities of the heparins were compared by ANOVA with Bonferroni post-hoc test using Origin 8.0 software (OriginLab).

### In vivo Antithrombotic Activity

Venous antithrombotic effects of the heparins were assessed by inducing formation of thrombus in mesentery veins of mice and then following their evolutions using an intra-vital fluorescence-microscope (Zeiss; Oberkochen, Germany), as described elsewhere. 13 C57BL/6 mice (∼12 g, both sexes) were anesthetized via intra-peritoneal with 2,2,2-tribromoethanol (body weight × 18 + 20 µL) from Sigma-Aldrich and then 20 µL of 0.1% Rhodamine 6G (Sigma-Aldrich) and different doses of each HOI, HBL and HPI (0.25, 0.5, 1.0 and 2.0 mg kg ^-1^ ) were injected in the retro-orbital plexus of the animals. Thereafter, suitable mesentery veins (∼200 µm diameter) were isolated after laparotomy and carefully positioned under the microscope objective in an apparatus with continuous flow of saline. Thrombus was induced by laying a filter piece (3 × 1 mm) soaked with 8% ferric chloride (Sigma-Aldrich) over the selected vein during 1 minute and then monitored for 60 minute or up to its complete occlusion (blood-flow interruptions longer than 30 second). The in vivo antithrombotic assays described above were performed by following in a strict manner the institutional guidelines for animal care and experimentation of our institution (Federal University of Rio de Janeiro).

## Results and Discussion

### Heparins from Different Sources have Distinct Molecular Sizes

**Fig. 2:**
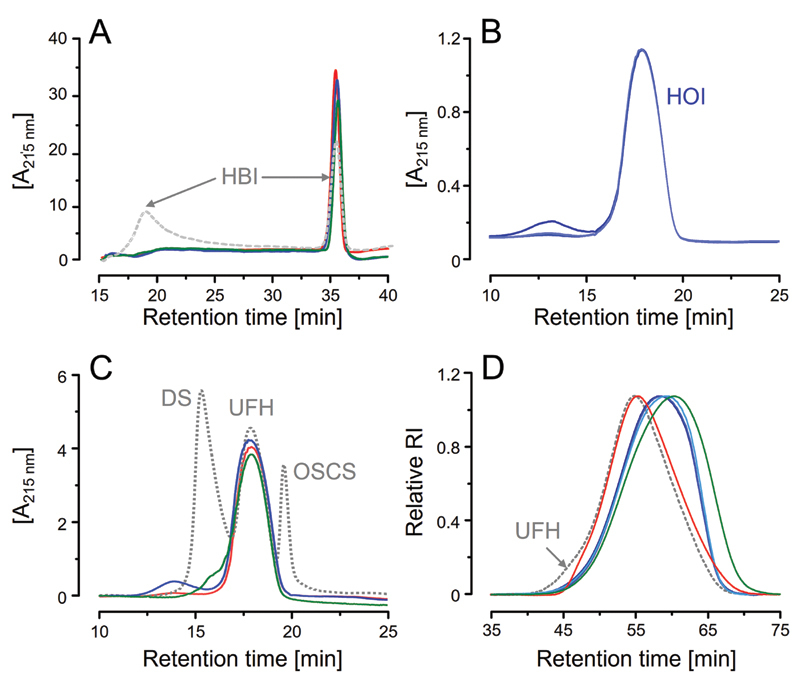
Chromatographic analysis. (A) Hi-resolution anion-exchange chromatograms show that HOI, HBL and HPI are composed by single populations of heparin chains (solid lines in blue, green and red, in all panels) whereas HBI is an heterogenous mixture of two heparin types (dashed gray line). Analytical anion-exchange chromatograms of three batches of HOI (B) and of one batch of each HOI, HBL and HPI and a mixture containing dermatan sulfate (DS), 6 ^th^ International Heparin Standard (HPI) and oversulfated chondroitin sulfate (OSCS; all depicted as a continuous dotted gray line). (D) Analytical size-exclusion chromatograms of HOI (3 batches), HBL, HPI and USP heparin molecular-weight calibrant (UFH; dotted gray line).

The use of chromatography methods for analyses of heparins date back to the 1960's decade. 16 Besides employed to assess anionic strength, purity and molecular size, recently we demonstrated that strong anion-exchange resins are able to evaluate whether pharmaceutical preparations have homogenous (a single population of heparin chains) or heterogenous (more than one population) compositions. 13 14 Different from HBI, which is clearly composed by two distinct heparins, both HOI, HBL and HPI contain a single and homogenous population of heparin chains ( Fig. 2A ). Therefore, HBI is still the only pharmaceutical heparin with heterogenous composition thus far, and, for this reason and their extensive compositional particularities will not be approached here.

As observed in the analytical anion-exchange chromatograms, the three baches of HOI have strictly coincident anion strength profiles and, except for the presence of a minor non-identified impurity in one of them, they are quite homogenous and pure ( Fig. 2B ). When analyzed following the recommendations for detecting glycosaminoglycan-based contaminants of heparin monographs of the United States, European and Brazilian Pharmacopeias (USP, EP and BP, respectively) ( Fig. 2C ), we observed that besides having similar anionic strengths, the pharmaceutical preparations of HOI, HBL and HPI analyzed here are devoid of impurities or contaminations with dermatan sulfate (DS) and oversulfated chondroitin sulfate (OSCS), except for the presence of trace amount of DS in HBL. 17 18 19

Another physical-chemical parameter analyzed by a chromatography technique was the molecular size of the heparins. Chromatograms obtained using analytical size-exclusion columns (SEC) showed that HOI and HBL have weight average molecular weight ( *M _w_* ) and number average molecular weight ( *M _n_* ) close to each other but significantly lower than HPI ( Fig. 2D , Table 1 ). Both HOI and HBL did not attend the molecular size recommended by USP and have contrasting heparin mass ( *M* ) > 24 kDa and *M* 8–16 kDa/M 16–24 kDa ratio values. 17 Otherwise, previous assessments on molecular size of HOI, HBL and HPI reported higher *M _w_* values, ranging out of USP specifications, for the three heparins. 20 Considering that current compendial parameters were stipulated for HPI preparations, with the eventual introduction of HOI as new pharmaceutical heparin product, the pharmacopeias should pay special attention to molecular size requirements.

**Table 1:** Average weight molecular weight ( *M _w_* ), number average molecular weight ( *M _n_* ), proportions of heparin mass ( *M* ) lower than 24 kDa and *M* 8→16 kDa / *M* 16→24 kDa ratio of HOI, HBL and HPI

	* M _w_* (Da)	* M _n_* (Da)	M > 24 kDa (% of total)	*M* 8→16 kDa / *M* 16→24 kDa
HOI a	13,670 ± 290	10,780 ± 210	8.1 ± 0.6	2.7 ± 0.1
HBL b	12,120 ± 210	9,570 ± 430	6.0 ± 0.6	3.3 ± 0.2
HPI c	16,540 ± 940 e	13,700 ± 130 e	13.3 ± 1.7	1.5 ± 0.3
USP Parameters d	15,000–19,000	*nd*	< 20	> 1

a Analysis of three batches.

b Three analysis of a single batch.

c Analysis of six batches.

d See USP [14].

e Significantly higher than HBL and HOI ( *p* < 0.05; ANOVA with Bonferroni post hoc test).

### NMR Spectra Revealed Variations among the Monosaccharide Compositions of the Heparins from Different Sources

**Fig. 3:**
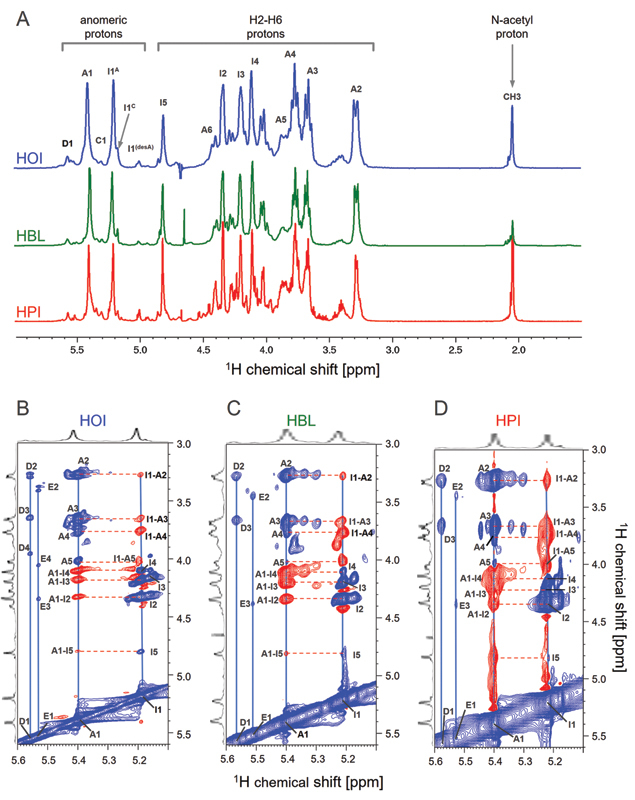
^1^ H NMR spectra reveal structural similarity among heparins from different sources. (A) 1D ^1^ H spectra (6.0–1.0 ppm) of HOI (blue), HBL (green) and HPI (red). Annotations in the panel indicate signals of the anomeric protons of α-Glc *N* ,6-diS (A1), α-Glc *N* S (C1); H1 (I1) and H5 (I5) of α-IdoA2S; and CH _3_ of the *N* -acetyl group from α-Glc *N* . (B–D) Phase-sensitive ^1^ H- ^1^ H TOCSY spectra of HOI, HBL and HPI differentiates signals of intra-residue spin systems (in blue) from those of inter-residue ROEs (in red). The anti-phase and in-phase connections are indicated by the horizontal red dotted and the blue vertical lines, respectively. The most intense anti-phase signals show the linkage to the subsequent sugar residue. Spin systems D and E correspond to β-GlcA linked to α-Glc *N* ,6di-S or α-Glc *N* ,3,6tri-S, respectively.

1D ^1^ H NMR spectra acquired for HOI, HBL and HPI are remarkably coincident ( Fig. 3A ), presenting signals with similar intensities and chemical shifts. The only noticeable difference seen on the spectra was the intensity of the signals at 2.06 (CH _3_ ) from *N-* acetyl groups (HPI > HOI > HBL), which indicates different amounts of *N-* acetylated α-D-glucosamine (α-Glc *N* ac) units in each heparin. 1D ^1^ H NMR spectra of the three batches of HOI are nearly the same (data not shown), which confirms the remarkable “between-batches” similarity of pharmaceutical heparins previously observed for spectra of more than 700 commercial batches of HPI analyzed by our group along the past 15 years. 10

Connections between the different α-D-glucosamine and hexuronic acid units component of HOI, HBL and HPI were investigated by using phase-sensitive 2D ^1^ H- ^1^ H TOCSY (phase-TOCSY) spectra. These spectra allow us to correlate protons of the same monosaccharide unit via scalar coupling ( *J* , correlation via chemical bond), as well as between adjacent units via dipolar coupling (NOE, correlation via spatial proximity). 21 The phase-TOCSY spectra of HOI, HBL and HPI ( Fig. 3B-D ) were similar to each other, which corroborate the structural resemblance observed on the 1D ^1^ H NMR. In-phase signals (in blue) are attributed to the correlations of protons belonging to the same residue (scalar coupling) and are indicated by blue vertical lines. For example, the hydrogen/proton linked to the carbon 1 (H1) of the β-D-glucuronic acid (β-GlcA) acid (assigned as D1) connects to H2 and H3 from the same sugar unit (assigned as D2 and D3). The same observation extends to the α-Glc *N* and α-Iduronic acid (α-IdoA) units, which in turn resonates in a more complex region of the spectra.

The anti-phase signals (in red) allowed to determine the coincidence of the connections between the monosaccharide unit's component of HOI, HBL and HPI. For example, we can ensure that their α-Glc *N* ,6-diS units (unit A) binds to α-IdoA2S (unit I), as indicated by the horizontal dashed red lines on the spectra, similarly in the three types of heparins ( Fig. 3B-D ). Phase-TOCSY spectra has shown to be particularly useful to clarify structural aspects of heparin. 8 Therefore, both 1D ^1^ H NMR and 2D ^1^ H- ^1^ H phase-TOCSY demonstrated that except for differences in the α-Glc *N* Ac content, HOI, HBL and HPI are structurally similar to each other.

**Fig. 4:**
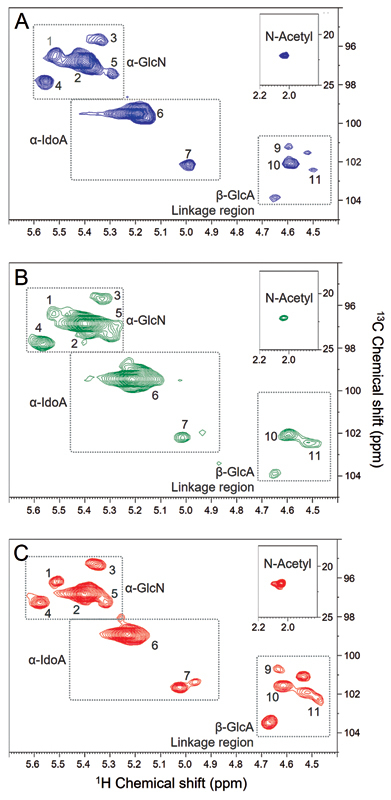
2D ^1^ H- ^13^ C HSQC NMR spectra of HOI (panel A in blue), HBL (panel B in green) and HPI (panel C in red). The numbers in the panels refer to the anomeric signals of the structures described in Table 2. The dotted squares in the Panels enclose the regions of the spectra containing the anomeric signals of α-D-glucosamine (α-GlcN), α-L-iduronic acid (α-IdA) and β-D-glucuronic acid (β-GlcA) + units of the linkage region and CH3 of the *N* -acetyl group (insert).

To investigate the structure of these different heparins in further detail, we acquired 2D ^13^ C- ^1^ H HSQC spectra, which have been increasingly employed to identify and quantify minor constituents of heparin preparations. 11 12 13 22 23 Signals attributed to anomeric protons of α-Glc *N* , α-IdoA and β-GlcA and from the linkage region to the protein core are enclosed by rectangles in the HSQC spectra of HOI, HBL and HPI shown in Fig. 4 . The structure, anomeric ^1^ H and ^13^ C chemical shifts (ppm) and quantifications of the different unit's constituent of each heparin, as well as the quantity of substitutions (sulfations or acetylation), are listed in the Table 2 .

**Table 2:** Proportions of the constituent units of HOI, HBL and HPI based on 2D ^1^ H/ ^13^ C HSQC spectra

Signal number a	Structure c	Chemical shift ( ^1^ H/ ^13^ C)	HOI ( *n* = 3)	HBL ( *n* = 1)	HPI f ( *n* = 8)
A) α-D-glucosamina b					
1	**α-Glc*N*,3,6-triS** -(?)	5.52/98.1	9.3 ± 1.8	3.5	5.5 ± 0.28
2-a	**α-Glc*N*,6-diS** -[α-IdoA-2S]	5.40/97.4	66.9 ± 4.4	79.8	63.9 ± 2.36
2-b c	**α-Glc*N*Ac,6S** -[β-GlcA] d		3.4 ± 0.3	2.5	5.4 ± 1.48
3	**α-Glc*N*,6-diS** -[α-IdoA]	5.36/99.5	4.7 ± 0.9	3.6	7.3 ± 1.44
4	**α-Glc*N*,6-diS** -[β-GlcA]	5.58/99.5	11.8 ± 3.3	6.9	11.0 ± 2.55
5	**α-Glc*N*S** -[α-IdoA-2S]	5.31/98.9	3.8 ± 0,42	3.7	7.50 ± 1.68
					
B) α-iduronic acid b					
6	**α-IdoA2S**	5.22/101.0	83.7 ± 0.2	88.4	78,3 ± 1,63
7	**α-IdoA** -[α-Glc *N,* 6-diS]	5.01/103.9	3.2 ± 0.2	2.9	2,3 ± 0,66
8	**α-IdoA**	4.95/103.7	0.5 ± 0.2	0.6	1,2 ± 0,23
	Σ α-IdoA		87.4	91.9	81.7
					
C) β-glucuronic acid b					
9	**β-GlcA** -[α-Glc *N* S,3,6-triS]	4.63/102.9	0.8 ± 0.2	0,7	2,1 ± 0,70
10	**β-GlcA** -[α-Glc *N* Ac,6S]	4.52/104.2	2.9 ± 0.3	2,3	8,8 ± 2,70
11	**β-GlcA** -[α-Glc *N* ,6-dIS]	4.61/103.8	6.4 ± 0.4	4,0	7,4 ± 0,30
12	**β-GlcA2S** -[?]	4.72/102.7	2.5 ± 0.2	1,1	18,3 ± 2,09
	Σ β-GlcA		12.6	8.1	36,6
					
	*N* -sulfation e		96.5	97.5	95.2
	6-sulfation		80.0	87.0	72.2
	3-sulfation		9.3	3.5	5.2
	2-sulfation		83.7	88.4	75.3
	*N* -acetylation		3.4	2.5	5.5

a See the rectangles in the panels of Fig. 4 .

b Results expressed as percentage of the total α-D-glucosamine or hexuronic acid units (α-L-iduronic + β-D-glucuronic acids).

c Reported structures in bold and the subsequent units in brackets.

d Proportions of the α-Glc *N* Ac residues were calculated by integrating H2/C2 signals at 3.86/56.2 ppm and subtracted from the anomeric signal representing both α-GlcNAc and α-GlcNS.

e Percentage of units bearing each substitution.

f Eight batches of HPI from two different suppliers (four of each).

Although the proton NMR analyses have revealed a notable structural equivalence, we found some clear differences among the heparins from different tissues on the HSQC spectra. Analyses of 8 batches of HPI from two different suppliers (four of each) showed variations much smaller than those among the distinct types of heparins, and thereby these differences cannot be attributed to “within-batches” variations. HBL presents more uniform structure, being mostly composed of α-Glc *N* ,6-diS-α-IdoA-2S disaccharides, this is reflected in its high *N* -, 2- and 6- sulfation but low β-GlcA and *N* -acetylation contents in comparison with the other heparins. HPI showed more heterogenous disaccharide composition and HOI an intermediary heterogeneity. Another relevant structural feature regards to the α-Glc *N* ,3,6-triS content, which is a pivotal component of the pentasaccharide sequence responsible for the binding with AT, and thus directly corelated with the anticoagulant activity of the heparins 24 ; the proportion of this minor but essential component varies among the tree heparins (HOI> HPI > HBL). As seen on the 1D ^1^ H spectra, the *N-* acetylation content of the heparins also vary (HOI > HPI > HBL), as shown in the inserts in the panels of Fig. 4 . In conclusion, HOI, HBL and HPI indeed have similar structures, as previous reported elsewhere, 20 25 26 27 but with noticeable differences among some of their minor constituents, especially in the sulfation pattern, *N-* acetylation content and proportion of α-Glc *N* ,3,6-triS units.

Notwithstanding HSQC certainly is a robust and reliable tool for heparin structural determination, quantification of part of the α-Glc *N* units sulfated in position 6 can be tricky due to signals overlapping on the spectra; furthermore, it is difficult to distinguish *N* -sulfated and *N* -acetylated αGlcN units by their HSQC anomeric cross-peaks, which thus requires integration of the C2/H2 signals. Accordingly, we also investigated the quantities of these and other units through chromatography analyses of disaccharides produced by digestion with heparinases.

### SAX-HPLC Disaccharide Analyses Confirmed the Structural Differences among Heparins from Different Sources

**Fig. 5:**
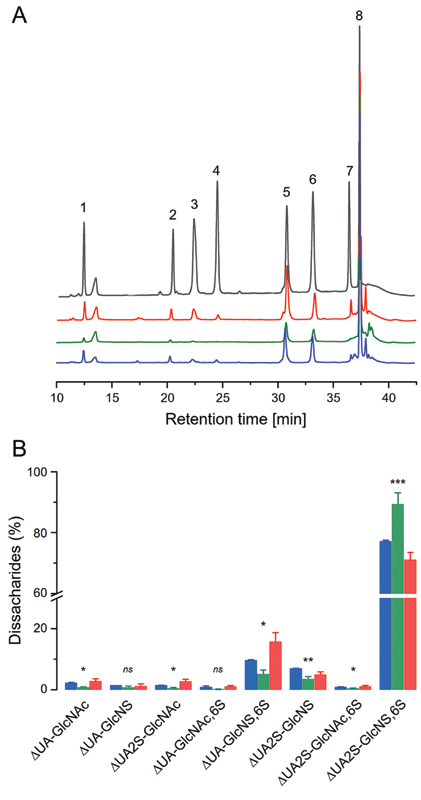
SAX-HPLC disaccharide analyses reveal differences among heparins obtained from different sources. (A) Chromatograms of the disaccharides formed by digestion of HOI, HBL and HPI (in blue, green and red, respectively, in all panels) with a mixture of heparinases I, II and III and of a mixture of heparin disaccharides standard (in black): 1) ∆UA-Glc *N* Ac; 2) ∆UA-Glc *N* S; 3) ∆UA2S-Glc *N* Ac; 4) ∆UA-Glc *N* Ac6S, 5) ∆UA-Glc *N* S6S; 6) ∆UA2S-Glc *N* S; 7) ∆UA2S-Glc *N* Ac6S; and 8) ∆UA2S-Glc *N* S6S. (B) Quantifications of the analyses of three chromatograms of each heparin: * HBL ≠ HPI; ** HOI ≠ HBL; *** HOI ≠ HBL ≠ HPI; and *ns* = non-significant differences (ANOVA with Bonferroni post hoc test).

Chromatography analyses (SAX-HPLC) of products formed by digestion of HOI, HBL and HPI, with a mixture containing the lyases heparinases I, II and III, allowed the identification and quantification of eight distinct disaccharide units, by comparing their retention times with those of a mixture of heparin disaccharides standards ( Fig. 5A ). As expected, the preponderant disaccharide found in the three heparins was the ∆UA2S-Glc *N* S,6S, which corresponds to the signal 2 on the HSQC spectra (see Fig. 4 and Table 2 ). The quantification of the peaks relative to the different disaccharide units ( Fig. 5B and Supplementary Table S1 (online only)) demonstrates that HBL certainly has a more homogenous disaccharide composition, whereas HPI is the more heterogenous (HBL > HOI > HPI). Analyses of 6 batches of HPI from two different suppliers (three of each) showed variations much smaller than those among the distinct types of heparins, as already reported in the quantifications based on the HSQC spectra ( Table 2 ). However, although we have detected minor differences in the disaccharide compositions of HOI, HBL and HPI, none of them can be assertively correlated to their different anticoagulant activities.

Quantifications of the different units component of HOI, HPI and HBL on the HSQC spectra or by SAX-HPLC analyses showed the same pattern but with small differences in the proportion of some disaccharides; for example, the proportion of units bearing 6-sulfation presented smaller values in HSQC than SAX-HPLC quantifications, it is because 6-sulfation is underestimated on the NMR analysis, since we cannot quantify accurately some units. Another relevant difference regards to the proportion of *N* -acetylated units, due to the difficult in distinguishing *N* -acetylated and *N* -sulfated α-GlcN units on NMR spectra. On the other hand, SAX-HPLC analyses do not differentiate α-IdoA from β-GlcA, since the cleavage with heparinases forms an unsaturation at the non-reducing terminals of the disaccharides. Furthermore, SAX-HPLC analyses cannot identify IdoA–α-Glc *N* S,3,6-triS unit component of the pentasaccharide sequence binding to AT because this unit is not cleaved by the lyases. The use of NMR (HSQC) and enzymatic/chromatography (heparinases/SAX-HPLC) as complementary techniques to achieve more in-depth structural characterizations of heparins has already proposed elsewhere, 20 28 and thus should be considered as a suitable protocol for performing comparability studies of heparins.

### Heparin from Different Sources also Generate Distinct Fragmentation Maps

After determining the disaccharide compositions with SAX-HPLC, we performed SEC analyses with the products formed by digestion of HOI, HBL and HPI with heparinase I to draw their fragmentation maps. Such analyses allow to determine the sequence of the different disaccharides along the heparin chains and has been routinely employed in comparability studies of generic versions of the LMWH enoxaparin. 29 Such fingerprinting analysis is based on the specificity of heparinse I in cleaving glycoside bonds involving α-IdoA, remaining intact those with β-GlcA, which results in a mixture of oligosaccharides with distinct chain lengths (e.g., di-, tetra-, hexa-, octasaccharides) that can be identified and quantified by using an analytical SEC chromatography column. 30

**Fig. 6:**
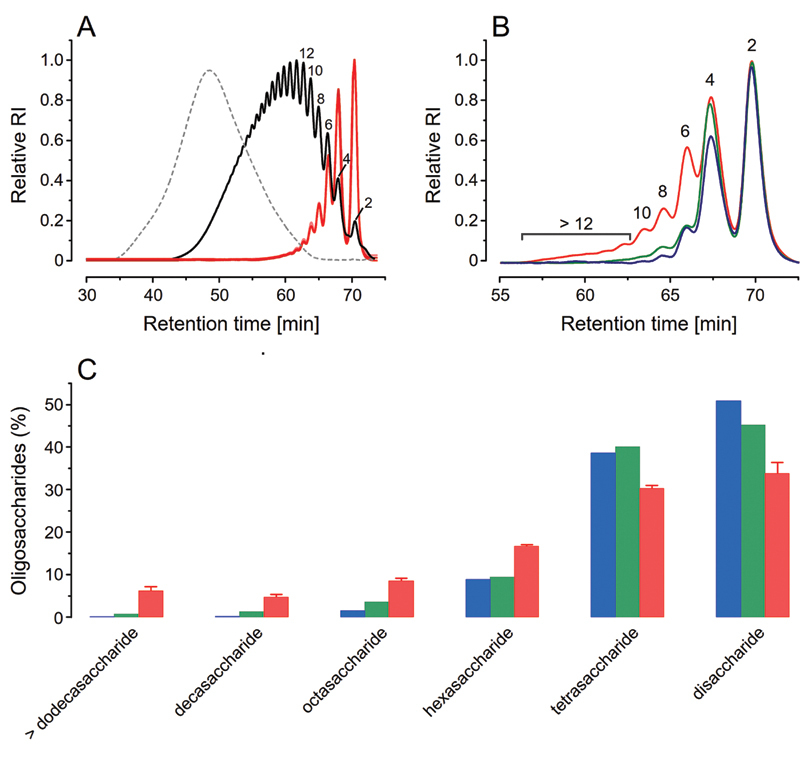
Heparins from different sources generate particular fragmentation maps. (A) Size-exclusion chromatograms of the oligosaccharides formed by digestion of three batches of HPI (red lines) with heparinase I; non-digested HPI (dashed gray line) and the 3 ^th^ International Standard for LMWH (solid black line), numbers in the panels indicate the degree of depolymerization of the oligosaccharides. (B) Chromatograms of the oligosaccharides formed by digestion of HOI, HBL and HPI (in blue, green and red, respectively, in all panels) with heparinase I. (C) Proportions of the oligosaccharides produced by each heparin type.

Considering that this analysis is mostly employed to compare LMWH preparations, we first assessed its precision and reproducibility by analyzing three different batches of HPI. SEC chromatograms of the three HPI batches after treatment with heparinase I were strictly coincident with each other ( Fig. 6A , solid reddish lines) and the depolymerization degree of the different oligosaccharides formed easily identified and quantified by comparing them with the chromatogram of the LMWH Molecular Mass Standard ( Fig. 6A , solid black line). The preponderant products were disaccharides (33.7%) and tetrasaccharides (30.2%), denoting regions containing exclusively α-IdoA or alternating β-GlcA/α-IdoA, respectively ( Supplementary Table S2 (online only)). Oligosaccharides with increased polymerization degree (36,1%) come from regions of the heparin chain with increasing amounts of β-GlcA units.

Then, we drawn the fragmentation maps of HOI and HBL, which yield chromatograms distinct both with each other and with those from HPI ( Fig. 6B ). Certainly, the differences between the maps of HOI and HBL are much more subtle than with those of HPI, which is clearly enriched in less depolymerized products (>hexasaccharide; Supplementary Table S2 (online only)) and thus must have more β-GlcA units unevenly distributed along their chains. Such increased content of β-GlcA (> 20%) and more complex composition of HPI were also found in the NMR and SAX-HPLC analyses; therefore, the variations quantified on the fragmentation maps of HOI and HBL must be useful for detecting minor differences between structurally similar heparins. Similarly, obtention of tetrasaccharide maps by analyzing products digested with heparinase II with liquid chromatography–mass spectroscopy (LC–MS) have also proven to be useful in differentiating HOI, HBL and HPI. 20

### A Balance between Different Physical-chemical Components Drives the Anticoagulant Activity of Heparins

**Fig. 7:**
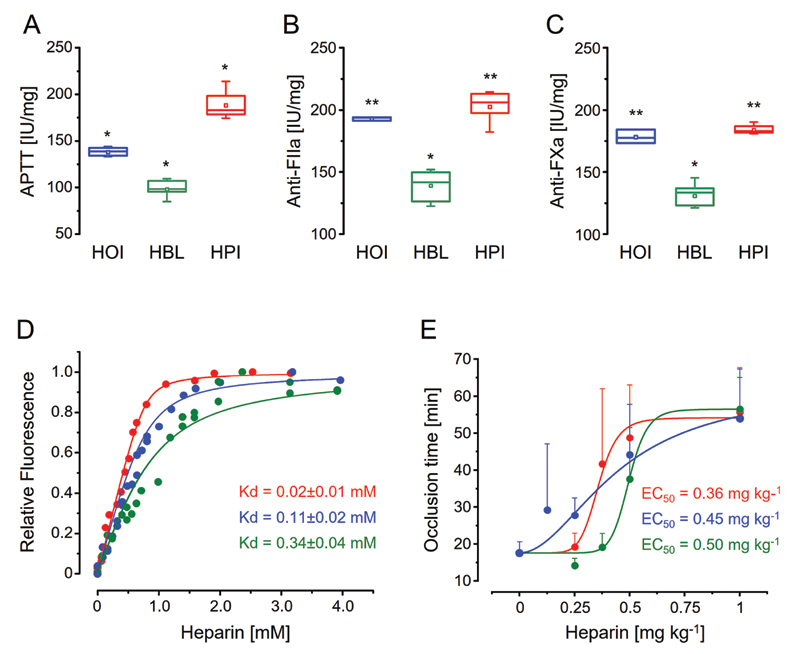
Heparins obtained from different source have distinct anticoagulant and/or antithrombotic activities. APTT (A), anti-FIIa (B) and anti-FXa (C) assays of HOI, HBL and HPI (in blue, green and red, respectively, in all panels). (D) Fluorometric analyses by titration of the different heparins with antithrombin performed to calculate their dissociation constants (K _d_ ). (E) Antithrombotic activities (EC _50_ ) of the different heparins in an animal model of venous thrombosis based on the determination of occlusion time of the jugular vein. * *p* < 0.05 and ** *p* > 0.05 (ANOVA with Bonferroni post hoc test).

Once we concluded the physical-chemical characterization of HPI, HOI and HBL, we approached their impact on the anticoagulant activity. Initially we test their effect on in vitro coagulation assays ( Fig. 7A-C ). We observed that HPI and HOI have similar activities on anti-FIIa and anti-FXa assays while HBL shows a significantly lower potency. On the other hand, in the APTT assays the three types of heparins maintained the same trend (HPI ≥ HOI > HBL) but with significantly different potencies, it is certainly because interactions with other components of anticoagulant cascade present in plasma but not in the purified systems employed in anti-FIIa and anti-FXa assays and/or nonspecific interactions with other plasma proteins, reducing bioavailability. 31 The low anticoagulant activity of HBL was previously reported and attributed mostly to its lower molecular size in comparison with HPI (12.1 kDa and 16.5 kDa, respectively). 8 However, HOI also has low molecular size (13.6 kDa) but anti-FIIa and anti-FXa activities similar to HPI ( Supplementary Table S3 (online only)) and, therefore, it remains to explain such a similar activity though the contrasting molecular size.

To clarify it, we investigated the affinity of the heparins with AT by determining dissociation constants (K _D_ ) with basis on changes in the intrinsic fluorescence resulting from their bindings with this serpin. 32 As expected, HPI has the highest affinity (K _D_ = 0.02 ± 0.01 mM); nevertheless, HOI presented K _D_ three times lower than HBL (0.11 ± 0.02 mM and 0.34 ± 0.04 mM, respectively) and thus a significantly higher affinity with AT ( Fig. 7D ). The reason for the different abilities of HOI and HBL in interacting to AT certainly is their different contents of α-Glc *N* ,3,6-triS (9.3% and 3.5%, respectively), which is a preponderant component of the heparin-AT binding pentasaccharide. 24 In fact, the diminished content of this trisulfated unit (1.1%) is attributed to the low affinity of HBI with AT and, consequently, for its reduced anticoagulant potency (∼120 IU mg ^-1^ ). 11 12 13 14 Therefore, we find that an eventual reducing in the anticoagulant activity of HOI resulting from its low molecular size might be compensated for a higher affinity to AT promoted by its increased α-GlcN,3,6-triS content, and thus the anticoagulant potency of different heparins is driven by a balance between different physical-chemical components, especially molecular size and fine-tuning composition.

Finally, the three heparins were tested in an animal model of venous thrombosis based on the time for occlusion of the jugular vein ( Fig. 7E ). Despite the high variability observed in the assays, we observed a correlation between anticoagulant activity and the antithrombotic effect (EC _50_ ) in the animal model (HPI ≥ HOI > HBL). Although the minor but noticeable differences found between the physical-chemical features and anticoagulant and antithrombotic activities of HPI and HOI corroborate that they are distinct drugs, these heparins could be approved by regulatory agencies for use as interchangeable anticoagulants. 33

## Conclusion

In the present study we evaluated the composition and physical-chemical characteristics of three different pharmaceutical preparations of unfractionated heparins employed in the past (HBL), in the present (HPI) and in development for future use (HOI). Although we have assessed only three batches of HOI and one of HBL, our results regarding both their physical-chemical and biological features are in accordance with information available in literature, 8 20 and thus they can be considered characteristic pharmacological preparations of each type of heparin. HBI was not included in the study due to its heterogeneous composition. The anticoagulant and antithrombotic effects of these heparins were also evaluated using in vitro assays and tests with an animal model of venous thrombosis. Such a “chemical-biological correlation” approach allowed us to conclude that the anticoagulant action of heparin relies on a synergic combination of physical-chemical factors. In particular we highlight the molecular size and presence of minor but biologically relevant units of α-Glc *N* ,3,6-trisulfated, which is a pivotal component of the AT binding region of the heparin.

Although a comprehensive assessment on physical-chemical features of HOI, HBL and HPI has already been reported, 20 here we further correlate it with the biological properties (anticoagulant and antithrombotic activities) of these different heparins. A practical aspect of our study is to orient the way these heparins should be consider for medical use, either as the same or distinct drugs. The results of the present study do not approach this point in detail but we suggest a variety of analytical approaches to differentiate these distinct heparin preparations. We emphasize the SEC analysis of heparinase I digested products, which yields fragmentation maps characteristic for each type of heparin. Another practical aspect we need to consider is the use of pharmaceutical heparins from alternative animal sources as start materials for production of new LMWH pharmaceutical products. Nowadays only HPI is used for preparation of LMWH but we need to investigate in detail the fragments obtained from new types of heparins to define their similarities with the current HPI-based gold standards. Some attempts have been reported for HOI but it requires investigations in further detail. 34 35 36

## Notes

**What is Known about this Topic?**

- Heparin is a biologic produced from mammal offal employed for more than a century as the lead anticoagulant drug.
- Pharmaceutical preparations from different animal sources usually have distinct physical-chemical features and anticoagulant activities.
- However, even heparins with similar chemical composition may have distinct anticoagulant potencies.
- Such distinct potencies are attributed to differences in the molecular size.

**What does this Paper Add?**

- We demonstrate that structurally similar heparins from porcine (HPI) and ovine (HOI) mucosa and bovine lung (HBI) actually have minor but relevant compositional differences.
- HOI and HBL have both reduced molecular sizes, however, HOI have anticoagulant activity similar to HPI whereas HBL is significantly less potent.
- The increased activity of HOI possibly results from an improved affinity to antithrombin (AT) promoted by the high content of a trisulfated glucosamine unit directly involved in heparin-AT binding.
- Notwithstanding putative similarities, heparins from different animal sources should always be treated as distinct drugs, with individual compendial and regulatory requirements.

## Footnotes

*
*These authors contributed equally as first authors.*
