# Supplementary material for: Anticoagulant Activity of Heparins from Different Animal Sources are Driven by a Synergistic Combination of Physical-chemical Factors
Source: TH Open. 2022 Oct 11;6(4):e309–22. doi: 10.1055/a-1946-0325 (PMC9593486; doi:10.1055/a-1946-0325)
Supplement: Supplementary file 1 — Supplementary Material [file 10-1055-a-1946-0325-s22070031.pdf]

Anticoagulant activity of heparins from different animal sources are driven by a synergistic combination of physical-chemical factors

Supplementary Information

Supplementary Table 1. Proportion (% of total) of the disaccharides formed by digestion of HOI, HBL and PPI with a mixture of heparinases I, II e III

**Disaccharide**[a]	**HOI (n=3)**	**HBL**[b] **(n=1)**	**HPI**[c] **(n=6)**
1) ΔUA-GlcNAc	2.19 ± 0.15	0.69 ± 0.29	2.73 ± 0.88
2) ΔUA-GlcNS	1.31 ± 0.02	0.70 ± 0.45	1.12 ± 0.80
3) ΔUA2S-GlcNAc	1.34 ± 0.14	0.40 ± 0.27	2.70 ± 0.72
4) ΔUA-GlcNAc,6S	0.77 ± 0.42	0.17 ± 0.11	0.94 ± 0.44
5) ΔUA-GlcNS,6S	9.58 ± 0.20	5.01 ± 1.42	15.70 ± 2.99
6) ΔUA2S-GlcNS	6.91 ± 0.07	3.39 ± 0.94	4.85 ± 1.00
7) ΔUA2S-GlcNAc,6S	0.78 ± 0.14	0.27 ± 0.27	0.94 ± 0.38
8) ΔUA2S-GlcNS,6S	77.13 ± 0.41	89.36 ± 3.75	71.01 ± 2.43
N-sulfatation	94.93 ± 0.56	98.46 ± 0.84	92.68 ± 2.03
6-sulfatation	88.25 ± 0.34	94.82 ± 1.95	88.60 ± 2.25
2-sulfatation	86.15 ± 0.34	93.42 ± 2.28	79.50 ± 1.54
N-acetylation	5.07 ± 0.56	1.54 ± 0.94	7.32 ± 2.03

[a]Numbers used to identify the disaccharides on the chromatograms of the Fig. 5.

[b]Values from three chromatograms of the single HBL sample.

[c]Three batches each from two suppliers.

Supplementary Table 2. Proportion (% of total) of the oligosaccharides formed by digestion of HOI, HBL and HPI with heparinase I.

**Oligosaccharide**	**HOI (n=1)**	**HBL[b] (n=1)**	**HPI (n=3)**
> dodecasaccharide	0.11	0.68	6.11 ± 1.1
decasaccharide	0.16	1.25	4.68 ± 0.7
octasaccharide	1.50	3.52	8.53 ± 0.6
hexasaccharide	8.85	9.35	16.69 ± 0.3
tetrasaccharide	38.56	39.99	30.24 ± 0.7
disaccharide	50.81	45.19	33.75 ± 2.6

Supplementary Table 3. Anticoagulant activity of HOI, HBL and HPI determined with APTT, anti-FIIa and anti-FXa assays. Results (mean ± SD) expressed in IU mg^{-1}.

	APTT	Anti-FIIa	Anti-FXa
HOI	138.6 ± 4.1	193.0 ± 1.6	178.3 ± 5.5
HBL	98.6 ± 8.2	139.3 ± 11.8	131.1 ± 8.7
HPI	188.6 ± 17.4	202.8 ± 11.3	184.2 ± 4.1
